# Case Report: Refractory *Listeria innocua* Meningoencephalitis in a Three-Year-Old Boy

**DOI:** 10.3389/fped.2022.857900

**Published:** 2022-05-19

**Authors:** Yi Liao, Lingling Liu, Hua Zhou, Feng Fang, Xinglou Liu

**Affiliations:** Department of Pediatrics, Tongji Hospital, Tongji Medical College, Huazhong University of Science and Technology, Wuhan, China

**Keywords:** *Listeria innocua*, meningoencephalitis, hydrocephalus, blood culture, infection

## Abstract

*Listeria innocua* is widely distributed in the environment and food and is considered a non-pathogenic bacterium for both humans and animals. To our knowledge, only a few cases of *L. innocua* infection in humans and ruminants have been reported. Moreover, there has been no report on human *L. innocua* meningoencephalitis. Here, we report a case of severe refractory meningoencephalitis in a three-year-old boy after infection with *L. innocua*. The child’s first symptoms were a runny nose, high fever, and rashes, which quickly progressed to unconsciousness and convulsions. The initial analysis of cerebral spinal fluid revealed remarkably elevated protein levels and increased white blood cells count. The blood culture of the patient in the early stage was positive for *L. innocua.* In addition, his brain imaging tests were observed dynamically, and the result showed a speedy progression from multiple intracranial abnormal signals to hydrocephalus and interstitial cerebral edema. After receiving antibiotics and symptomatic treatment for nearly 3 months, the patient’s condition improved markedly. However, he still had residual complications such as hydrocephalus. Although *L. innocua* is considered harmless, it can still cause disease in humans, even severe meningoencephalitis, with rapid progression and poor prognosis. Early discovery, diagnosis, and treatment are necessary to elevate the survival rate and life quality of those patients. Antibiotics should be used with sufficient duration and dosage. Cephalosporins are not suitable for the treatment of *L. innocua* meningoencephalitis and penicillin antibiotics are preferred for children. The presentation of this case will help to expand our knowledge of *Listeria* infections and provide a potential candidate for pathogens causing severe childhood central nervous system infection.

## Introduction

*Listeria* species are small rod-shaped gram-positive bacteria, ubiquitous and widespread in the environment. *Listeria* infections are mainly due to ingestion of contaminated food, such as fruits, raw meat and fish, milk, uncooked vegetables, etc., (1). *Listeria*-related diseases are usually observed in humans and ruminants, which can also be occasionally found in pigs, rabbits, poultry, and other animals. Until now, 21 species of *Listeria* were recognized (2), of which species *Listeria monocytogenes* is the major human pathogen that causes highly fatal opportunistic foodborne infection in pregnant women, neonates, the elderly, and immunocompromised patients. Besides *L. monocytogenes* can also lead to infections in many vertebrate species, including birds. Whereas, as another well-known pathogenic species, *Listeria ivanovii* infection is almost unique to ruminants (3), with rare cases of infection reported in humans (4). According to a series of recent case reports, two principal clinical forms of listeriosis are sepsis and neurolisteriosis, accounting for over 80% of cases (5, 6). In those neurolisteriosis cases, meningoencephalitis takes the major part. In contrast to *L. monocytogenes and L. ivanovii, Listeria innocua* does not readily cause disease in mammals and so was named “innocua” (7). So far, rare cases of *L. innocua* infection have been reported in humans (8–10) and ruminants (11, 12). Here, we describe a case of refractory *L. innocua* meningoencephalitis in a child, which may help pediatric professionals to further understand *Listeria* infections and provide new clues for clinical diagnosis and treatment.

## Case Description

**FIGURE 1:**
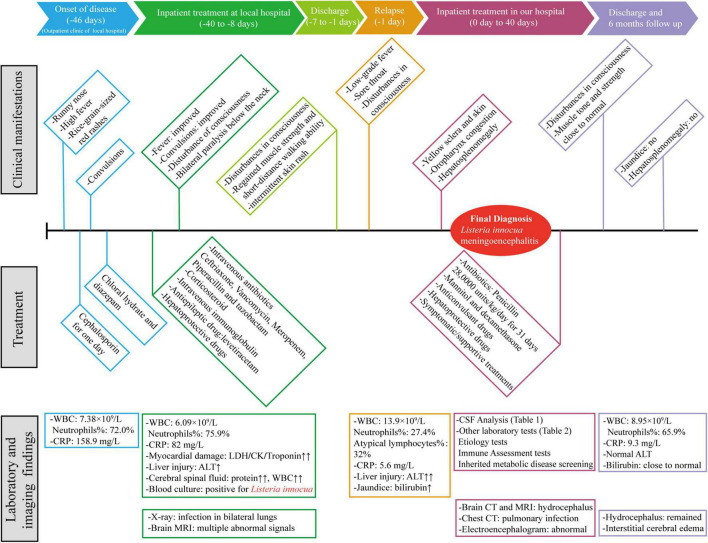
A timeline with relevant data from the patient episode of care. WBC, white blood cell; CRP, C-reactive protein; LDH, lactate dehydrogenase; CK, creatine kinase; ALT, alanine aminotransferase; CSF, Cerebral spinal fluid.

A three-year-old boy was admitted to our hospital on 16 April 2020. The first symptoms appeared 46 days ago (1 March 2020), manifested as a runny nose, high fever, and rice-grain-sized red rashes. Then, the patient visited the local hospital, and his laboratory findings showed a white blood cell (WBC) count of 7.38 × 10^9^/L (reference range: 4–12 × 10^9^/L) with high neutrophils percentage (72%) (reference range: 26.3–63.1%) and C-reactive protein level (CRP, 158.9 mg/L, reference range: < 0.5 mg/L). After receiving cephalosporin in the outpatient clinic for 1 day, the child started to have convulsions which manifested as loss of consciousness, staring eyes, foaming at the mouth, and twitching of the corners of the lip. His convulsions lasted for half an hour and were relieved after treatment with chloral hydrate and diazepam. Subsequently, he was transferred to the Pediatric Intensive Care Unit for further treatment on March 6. A timeline with relevant data from the patient episode of care is shown in Figure 1.

**FIGURE 2:**
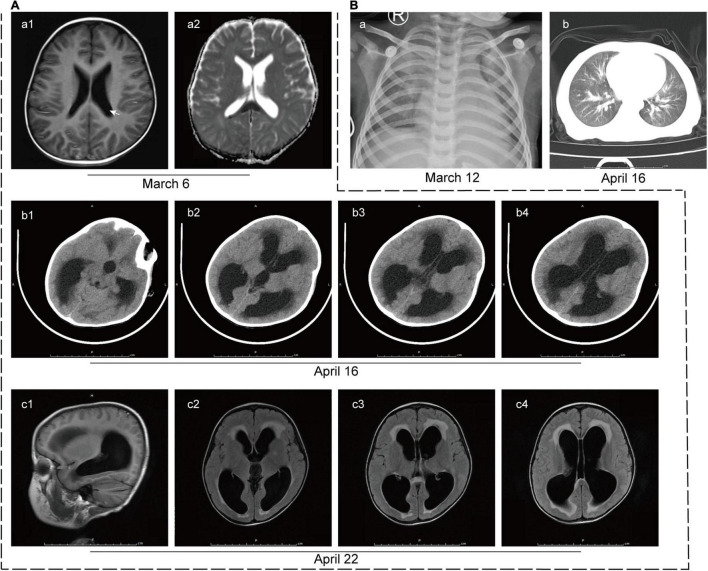
Imaging tests of the patient. **(A)** Imaging tests of the brain. (a) Brain diffusion-weighted MRI on 6 March 2020 revealed multiple intracranial abnormal signals with the slightly enlarged posterior horn of the lateral ventricle (white arrow). (b) CT scan of the brain on 16 April 2020 showed hydrocephalus and interstitial edema of white matter. (c) T2-Flair MRI images of the brain on 22 April 2020 showed multiple patchy shadows in the splenium of the corpus callosum, frontal lobe, and left basal ganglia, suggesting infectious changes. Hydrocephalus and interstitial edema were also observed. **(B)** Imaging tests of the lung. (a) The initial lung X-ray image of the patient on 12 March 2020 showed infection in the bilateral lungs. (b) CT scan of the lung on 16 April 2020 revealed patchy shadows on bilateral lungs, suggesting infection.

At this time, initial laboratory and imaging tests were performed. Full blood count testing showed a WBC count of 6.09 × 10^9^/L with a neutrophil ratio of 75.9%. CRP level remained high (82 mg/L). The lactate dehydrogenase, creatine kinase and troponin levels were 3,887 U/L (reference range: 120–300 U/L), 2,090 U/L (reference range: ≤ 190 U/L) and 518 pg/ml (reference range: < 1.9 pg/ml), respectively, suggesting myocardial damage. Mild liver injury was also noted, with an alanine aminotransferase (ALT) level of 95 U/L (reference range: ≤ 41 U/L). Cerebral spinal fluid (CSF) analysis showed that CSF protein level was remarkably elevated and WBC count was also increased, of which lymphocytes account for the majority (Table 1). It is worth noting that the blood culture of the patient was positive for *L. innocua*. In addition, the chest X-ray image showed infection in bilateral lungs (Figure 2B-a) and brain diffusion-weighted MRI showed multiple intracranial abnormal signals with the slightly enlarged posterior horn of the lateral ventricle (Figure 2A-a).

**TABLE 1:** Lumbar puncture and CSF analysis findings.

Cerebrospinal fluid		Results		
		March 6	March 30	April 16
Appearance		Yellowish and clear	Clear	Yellowish and clear
Protein level (mg/L)		3,057↑	747↑	829↑
Glucose (mmol/L)		4.05	3.68	4.23
Chloride (mmol/L)		120	117	118
Lactate dehydrogenase (U/L)		973↑	58↑	27
Stains and smears		Negative	Negative	Gram stain (-), India ink stain (-), Acid-fast bacilli smear (-)
Bacterial culture		Negative	Negative	Negative
White blood cell	Counts (× 10^6^/L)	145	7	2
	Neutrophils (%)	26	-	-
	Lymphocytes (%)	74	-	-

The patient was treated with intravenous antibiotics (ceftriaxone, vancomycin, meropenem, piperacillin, and tazobactam), corticosteroid, intravenous immunoglobulin, antiepileptic drug (levetiracetam), and hepatoprotective drugs (reduced glutathione and bicyclol). After the above treatment, his fever and convulsions improved, and the levels of biomarkers for myocardial and liver injury were gradually decreased. However, he still had a disturbance of consciousness accompanied by bilateral paralysis below the neck.

On 30 March 2020, CSF analysis was repeated and both CSF protein level and WBC count were dropped (Table 1). Then the patient was transferred from the local intensive care unit to a general ward. Subsequently, his intravenous administration was stopped, and he was discharged from the hospital a week later. Though disturbances remained in consciousness, he regained his muscle strength and can have a short walk. The patient still had intermittent skin rash manifested as a red needle-like rash without itch on the lower limbs and back which disappeared after oral administration of antihistamines.

About 1 week later (15 April 2020), the patient got a fever again, which manifested as low-grade fever and can retreated spontaneously, accompanied by a sore throat with a large white discharge in the oropharynx. Full blood count testing showed a WBC count of 13.9 × 10^9^/L with a neutrophil ratio of 27.4%, while the proportion of atypical lymphocytes reached 32%. CRP level was 5.6 mg/L. Liver injury reappeared with higher ALT level (245 U/L) and jaundice (total and direct bilirubin of 95.9 and 79.8 μmol/L, respectively) (reference range: ≤ 26 and ≤ 8 μmol/L, respectively). Because of his high atypical lymphocyte rate, an Epstein-Barr virus antibody test was also carried out. The result showed positive in viral capsid antigen (VCA)-IgG and nuclear antigen (EBNA)-IgG, which indicated that the patient had a past Epstein-Barr virus infection, and it is not related to the current liver injury.

For further diagnosis and treatment, the patient was admitted to our hospital on 16 April 2020. The initial physical examination revealed visibly yellow sclera and skin, congestion and large secretions in the oropharynx, and hepatosplenomegaly (7 and 5 cm below the costal margin, respectively, both edges were hard). CT scans of the brain (Figure 2A-b) and the chest (Figure 2B-b) were performed to show the hydrocephalus and pulmonary infection. The result of the long-term video electroencephalogram showed high amplitude delta activity with intermittent spike-and-slow-wave discharges over the left frontal-anterior temporal region. Therefore, intracranial infection and icteric hepatitis were two major problems to be solved urgently at that time. To better investigate the cause of the disease, further laboratory (Tables 1, 2) and imaging (Figure 2A-c) tests were performed.

**TABLE 2:** Further laboratory examinations of the patient.

Examinations	Test details	Results
**Etiology**		
- Epstein-Barr virus	Antibody test	VCA-IgG (+), EA-IgG (+), EBNA-IgG (+), VCA-IgM (-)
	Nucleic acid	Whole blood (-), plasma (-)
- The nine pathogens of respiratory tract infection (IgM antibodies)	Legionella pneumophila serogroup 1, Mycoplasma pneumoniae, Coxiella burnetii, Chlamydia pneumoniae, Adenovirus, Respiratory syncytial virus, Influenza A virus, Influenza B virus, Parainfluenza virus type 1/2/3	All negative
- Fungi	β-D-Glucan Assay	Negative
	Galactomannan assay	Negative
- SARS-CoV-2	Antibody test	IgG (-), IgM (-)
	Nucleic acid	Negative
- TORCH	Antibody test of IgM	All negative
- Hepatitis viruses	Antibody test for Hepatitis A, B, C, E	All negative
- Treponema pallidum	Antibody test	Negative
- Parvovirus B19	Antibody test	Negative
- Tuberculosis	T-SPOT.TB test	Negative
**Immune Assessment Tests**		
- Assays of circulating cytokines	-	IL-1β↑, IL-2R↑, IL-6↑, IL-8↑, IL-10↑, TNF-α↑
- Lymphocyte subset analysis	Cell count (cells/μl)/proportion (%)	Total T cells: 3,520/94.51↑ - CD4^+^ T cells: 658/17.68↓ - CD8^+^ T cells: 2,843↑/76.34↑ Total B cells: 21↓/0.56↓ NK cells: 160/4.3
- Immunoglobulin and Complement Test	IgA, IgG, IgM, C3 and C4	IgA↓, IgM↓
- Autoantibody test for autoimmune encephalitis, hepatitis, and systemic autoimmune diseases	Both blood and cerebrospinal fluid	All negative
**Inherited Metabolic Disease Screening**	-Lactic acid and pyruvate testing	In normal range
	-Amino acids blood test	No obvious abnormalities
	-Urine organic acids analysis	No obvious abnormalities

*VCA, viral capsid antigen; EA, early antigen; EBNA, EBV nuclear antigen; TORCH, stands for toxoplasmosis, rubella, cytomegalovirus (CMV), and herpes simplex virus (HSV).*

Combining the patients’ medical history and previous laboratory and imaging results, especially the blood culture positive for *L. innocua*, and ruling out infection from other pathogens, we consider a diagnosis of *L. innocua* meningoencephalitis (13). Based on the diagnosis, medical treatments were given as follows, antibiotics (penicillin, 280,000 units/kg/day, lasted for 31 days), mannitol, and dexamethasone to reduce cerebral edema, anticonvulsant and hepatoprotective drugs (reduced glutathione, magnesium isoglycyrrhizinate, and bicyclol). Other symptomatic and supportive treatments were also provided.

Brain imaging tests were reviewed at 2 and 4 weeks after treatment and the infectious lesions were partly absorbed. Repeated blood and cerebrospinal fluid cultures during and after treatment were negative for *L. innocua*. Nearly 1-month post-treatment, the patient had no fever, rash, or convulsions, and his physical examination showed reduced jaundice and hepatosplenomegaly (2 and 3 cm below the costal margin, respectively). Although he still had disturbances in consciousness, his muscle tone and strength were basically returned to normal levels. Full blood count testing showed a WBC count of 8.95 × 10^9^/L with a neutrophil ratio of 65.9%. CRP level was 9.3 mg/L. Liver injury was improved with ALT of 13 U/L and total and direct bilirubin were 38.3 and 34.3 μmol/L, respectively. The patient was then discharged home. In his subsequent follow-up, the patient showed no signs of jaundice, and his hepatosplenomegaly was back to normal, but hydrocephalus and interstitial cerebral edema remained.

## Discussion

As a member of the *Listeria* genus, *L. innocua* is widespread in the natural environment (such as oil and food). It is close to *L. monocytogenes*, an important etiological agent of listeriosis, but is generally considered non-pathogenic to humans. The major difference between the two species is that *L. innocua* is usually non-hemolytic. Recently, increasing isolates of atypical hemolytic *L. innocua* have been identified from different kinds of food, and they are also widely distributed (14, 15). Research has revealed that atypical hemolytic *L. innocua* clades also carry the *L. monocytogenes* pathogenic islands such as LIPI-1 and LIPI-3 (14, 16) and were able to invade human Caco-2 cells. Another study of *in vitro* and *in vivo* assays has confirmed that hemolytic *L. innocua* clades are virulent, can actively cross the intestinal epithelium, and spread to the liver and spleen, albeit to a lower degree than the *L. monocytogenes* (17). It is indicated that some clades of *L. innocua* also have a certain capacity to infect humans.

The first case of human *L. innocua* infection was published by *Perrin et al.* in 2003. It described a 62-year-old woman who suffered from fatal *L. innocua* sepsis (8). Thereafter in 2014, there were two reports of *L. innocua* infection. One case was an elderly woman with *L. innocua* meningitis (9). Though prompt diagnosis and antibiotic treatment were given, she was still complicated with obstructive hydrocephalus and eventually died of concurrent *Acinetobacter baumannii* pneumonia. The other case was an *L. innocua* infection in a 9-month-old boy (10). Before disease onset, the baby had developed hydrocephalus because of perinatal intraventricular hemorrhage and received a ventriculoperitoneal shunt when he was 40 days old. *L. innocua* were isolated from both CSF culture and ascites fluid of the infant. In the three above cases, the two adult patients were aged over 60 and had taken inhaled and oral corticosteroids, respectively, for several months prior to the infection which led to some immunodeficiencies. In the remaining infant case, he had received a ventriculoperitoneal shunt before the central nervous system infection, which might increase the risk of intracranial infection. However, the present report is the first case, to our knowledge, describing *L. innocua* meningoencephalitis in a child who has not undergone invasive surgery and has not received any corticosteroid drugs before the onset of the disease. Interestingly, the lymphocyte subsets analysis of this child showed a low B cell count, indicating that the patient had a certain degree of immunodeficiency after infection. Whether the occurrence of B cell reduction is inherent or caused by infection remained unclear.

Since central nervous system infection of *Listeria* is dangerous, progressing rapidly with high mortality, quick diagnosis and early effective treatment are important. Microbiological tests are the only way to confirm the diagnosis of *Listeria* meningoencephalitis. However, due to the low sensitivity of the CSF Gram stain, diagnosis is usually established by the culture of the bacteria from CSF or blood. According to a series of retrospective studies, the positive rates of blood and CSF culture in suspected neurolisteriosis cases were 61–64 and 41–83%, respectively (18, 19). In the present report, the diagnosis was based on clinical manifestations of the central nervous system, MRI results, and CSF pleocytosis, and was further established by culture of blood (13).

In consideration of antibiotic resistance, penicillin antibiotics are preferred for children to treat *L. innocua* meningoencephalitis, while cephalosporins are not suitable (1, 20). Carbapenems, aminoglycosides, sulfonamides, quinolones, or glycopeptide antibiotics might be considered when necessary (21). In the present case, the patient was not treated with appropriate antibiotics (such as penicillins) during the initial presentation, due to a lack of adequate knowledge of *L. innocua* by the local doctors. This might be one of the factors leading to relapse of symptoms and refractory infection in the patient. The head MRI of the patient showed abnormal infectious signals in the early stage, which speedily developed into hydrocephalus with interstitial cerebral edema. After nearly 3 months of treatment, the boy’s symptoms were partially improved, but his hydrocephalus still existed. The review of our experience in diagnosis and treatment suggests that early discovery, rapid diagnosis, and sufficient duration and dosage of antibiotics are crucial.

## Conclusion

Although uncommon, *L. innocua* can also infect humans, leading to serious disease and even death. Early diagnosis with proper treatment is the essential way to improve the prognosis. In the present case, the boy received a full course of antibiotic treatment, but it failed to reverse the rapid progress of central nervous system impairment. As a late complication of *L. innocua* meningoencephalitis, hydrocephalus points to a poor outcome, and its influence on the patient needs further observation and rehabilitation.

## Data Availability Statement

The original contributions presented in the study are included in the article/supplementary material, further inquiries can be directed to the corresponding author/s.

## Ethics Statement

Ethical review and approval was not required for the study on human participants in accordance with the local legislation and institutional requirements. Written informed consent to participate in this study was provided by the participants’ legal guardian/next of kin.

## Author Contributions

YL wrote the first draft of the manuscript. XL contributed to manuscript revision. All authors participated in the patient’s care and read and approved the submitted version.

## Conflict of Interest

The authors declare that the research was conducted in the absence of any commercial or financial relationships that could be construed as a potential conflict of interest.

## Publisher’s Note

All claims expressed in this article are solely those of the authors and do not necessarily represent those of their affiliated organizations, or those of the publisher, the editors and the reviewers. Any product that may be evaluated in this article, or claim that may be made by its manufacturer, is not guaranteed or endorsed by the publisher.
